# Distribution of serotypes, biotype, and antibiotic sensitivity of *Haemophilus influenzae* in nasopharyngeal carrier children in Lima, Peru

**DOI:** 10.17843/rpmesp.2025.424.14767

**Published:** 2025-11-20

**Authors:** Brayan E. Gonzales, Maria Pinedo-Bardales, Marcela Lopez-Briceño, Edison A. Rivera-Fernandez, David Durand, Erik H. Mercado, Faviola Valdivia, Theresa J. Ochoa

**Affiliations:** 1 Instituto de Medicina Tropical Alexander von Humboldt, Universidad Peruana Cayetano Heredia, Lima, Peru. Universidad Peruana Cayetano Heredia Instituto de Medicina Tropical Alexander von Humboldt Universidad Peruana Cayetano Heredia Lima Peru; 2 Laboratorio de Infecciones Respiratorias Agudas, Centro Nacional de Salud Pública, Instituto Nacional de Salud, Lima, Peru. Laboratorio de Infecciones Respiratorias Agudas Centro Nacional de Salud Pública Instituto Nacional de Salud Lima Peru; 3 Facultad de Medicina, Universidad Peruana Cayetano Heredia, Lima, Peru. Universidad Peruana Cayetano Heredia Facultad de Medicina Universidad Peruana Cayetano Heredia Lima Peru

**Keywords:** Hib Vaccine, Haemophilus influenzae, Colonization, Serotypes, Biotypes

## Abstract

After the introduction of the Hib (*Haemophilus influenzae* type b) vaccine, invasive infection caused by this bacterium markedly decreased; however, concern increased regarding the other typable and non-typable strains (NTHi). Prior colonization with this pathogen can become a source of invasive infection. This study aimed to determine colonization by *H. influenzae* in 888 healthy children under 24 months of age. Colonization was 4.1% (37 samples) using molecular methods (all NTHi) and 1.1% (10 isolates) by culture (8/10 biotype I and 2/10 biotype IV). A history of prematurity was associated with higher colonization in multivariate analysis. Of the isolates, 9/10 were resistant to sulfamethoxazole/trimethoprim, 3/10 to ampicillin and cefuroxime, 2/10 to azithromycin, and no resistance to ceftriaxone was observed. Understanding the carriage status of *H. influenzae* and local resistance patterns is useful for empirical antibiotic therapy in invasive infections where this bacterium is suspected.

## INTRODUCTION

*Haemophilus influenzae* is a Gram-negative coccobacillus that can cause infections such as otitis media, sinusitis, and pneumonia, mainly in children, the elderly, and immunocompromised patients ^1^. Its transmission primarily occurs through contact with respiratory droplets from carriers. Neonates can become infected through contact with infected genital secretions ^2^.

Colonization by *H. influenzae*, and other respiratory pathogens like *Streptococcus pneumoniae*, is common in young children. However, colonization can become a source of invasive infection. Before the vaccines against *S. pneumoniae* and *H. influenzae* type b (Hib), respiratory infections were the main cause of mortality in children <5 years old ^3^. Of the six serotypes of *H. influenzae* (a, b, c, d, e, and f), serotype b (Hib) was the main cause of meningitis in the pediatric population, before the introduction of the Hib vaccine in the 1990s ^4^. Likewise, there are non-encapsulated strains, called non-typable (NTHi). Although the role of the Hib vaccine in replacement is not clear, NTHi strains have become a growing cause of invasive disease ^1^.

Three decades after the introduction of the Hib vaccine worldwide (partially introduced in 1998 and universally in 2004 in Peru ^5^), the epidemiology of invasive *H. influenzae* disease has substantially changed ^1^, with a respective increase in other serotypes ^4^. Similarly, eight biotypes are used as epidemiological markers to study colonization and pathogenic strains. Biotype I is associated with meningitis in children and greater antimicrobial resistance; biotypes II and III with upper respiratory tract infections ^(6-8)^. Currently, the increasing resistance of *H. influenzae* to beta-lactams, especially penicillin, is a matter of concern ^9^^,^^10^.

Prevention and management strategies for *H. influenzae* must be based on surveillance studies. However, these studies are scarce in Peru. The objective of this study was to identify the main serotypes and biotypes of *H. influenzae* in children <24 months attending health facilities, as well as their antimicrobial resistance profiles and factors associated with colonization status.

**KEY MESSAGES**

Motivation for performing the study. Following the introduction of the Hib vaccine, invasive infections decreased, but concern over non-typable strains increased. This study sought to evaluate *H. influenzae* colonization in healthy Peruvian children.

Main findings. Colonization was 4.1% by molecular methods and 1.1% by culture, with all strains being non-typable. Biotype I predominated. Only 3/10 strains were resistant to ampicillin, and there was no resistance to ceftriaxone. Premature birth of the child was associated with increased colonization.

Implications for public health. The findings highlight the need for continuous surveillance and knowledge of local resistance to guide empirical therapy and prevention strategies.

## THE STUDY

### Study design

This multicenter cross-sectional study used data and samples from the previous study on *S. pneumoniae* carriers ^11^, which included 1000 healthy children aged 2-24 months seen in pediatric, healthy child check-up, and vaccination consultations at five national hospitals (Hospital Nacional Cayetano Heredia, Instituto Nacional de Salud del Niño Sede Breña, Hospital Nacional Edgardo Rebagliati Martins, Hospital Nacional Docente Madre Niño San Bartolomé, and Hospital Nacional Daniel Alcides Carrión) in Lima between 2018-2019. The same inclusion and exclusion criteria as the previous study were used, of which, only 888 samples belonged to participants who had consented to their use in future studies and had remaining samples.

### Sample collection, transport, and processing

In the previous study, nasopharyngeal samples were collected from participants under sterile technique with a Rayon swab (Puritan®), following the recommendations of the World Health Organization ^11^. The samples were transported in STGG (Skim milk, tryptone, glucose and glycerol) medium on ice, maintaining the cold chain and within 8 hours of their collection to the Pediatric Infectology Laboratory of the Universidad Peruana Cayetano Heredia ^11^. Then, a 200µL aliquot was stored in STGG at -80°C for future studies. This study used these stored samples, which were enriched in Brain Heart Infusion (BHI) supplemented with 5% of *Haemophilus Test Medium Supplement* (OXOID) for 4-6 hours at 37 °C in 5% CO2. The enriched culture was plated on chocolate agar with bacitracin under the previously described conditions for 24 hours ^12^.

### Microbiological and molecular identification of *H. influenzae*

*H. influenzae* isolates were identified by colony morphology, absence of hemolysis, Gram stain, catalase test, and requirement for factors X and V ^13^. Additionally, all nasopharyngeal swabs were evaluated by polymerase chain reaction (PCR) to detect the *Hpd* gene of *H. influenzae* (supplementary table 1).

### Microbiological biotyping and molecular serotyping

The biotypes of *H. influenzae* positive isolates were evaluated using the urease test, indole production, and ornithine decarboxylation ^14^. DNA from the isolates was obtained using Chelex®100, and a series of sequential PCRs were performed for serotyping. The first PCR detects the *Hpd* gene (*H. influenzae* confirmation), and confirmed strains proceeded to a PCR with six *primer* pairs to identify the capsular types (supplementary table 1), using reference strains as a positive control.

### Antibiotic susceptibility

Antibiotic susceptibility to ampicillin, amoxicillin/clavulanic acid, ampicillin/sulbactam, cefuroxime, ceftriaxone, cefotaxime, meropenem, levofloxacin, sulfamethoxazole/trimethoprim (SXT), azithromycin, and chloramphenicol was determined by the disk diffusion method. For the definitions of sensitivity (susceptible, intermediate, resistant), the standards of the Clinical and Laboratory Standard Institute (CLSI) published in document M100, 33rd Ed were used ^15^.

### Operationalization of variables

The previous study conducted a survey of the child’s caregiver to collect demographic and clinical information. A directed acyclic graph (DAG) was constructed to select variables potentially associated with *H. influenzae* colonization. These variables were sex, age in months, age group (≥2-<6, ≥6-<12, ≥12-<18, and ≥18-≤24 months), duration of breastfeeding in months, completed exclusive breastfeeding for 6 months, daycare attendance, living with other children <6 years old, sampling season (fall, winter, spring, and summer), premature birth, having used antibiotics prior to 3 months before sample collection, having been hospitalized up to 3 months prior to sample collection, presence of at least one mild respiratory pathology (mild cough, sneezing, rhinorrhea, fever between 38-38.5°C), and immunized with PCV13 (Pneumococcal conjugate vaccine 13).

### Statistical analysis

The description of the characteristics and comparison with carriage frequencies were performed using the Student’s t-test (for numerical variables with normal distribution), Chi-square (χ2), and Fisher’s exact test (for categorical variables). The prevalence ratio (PR) was estimated as a measure of association using Poisson regression with robust variances. Using epidemiological criteria, all variables potentially associated with *H. influenzae* colonization were selected in the multivariate adjusted model. A 95% confidence interval (95% CI) and a p-value <0.05 were considered statistically significant. All analyses were performed using the statistical package Stata/SE v.18.0.

### Ethical aspects

The study was registered in the Decentralized Research Information and Monitoring System (SIDISI, Spanish acronym) and evaluated by the Institutional Ethics Committee of the Universidad Peruana Cayetano Heredia. Only samples from participants who had consented to the future use of their sample in the initial pneumococcal carriage study were included.

## FINDINGS

Of the sample included, the distribution by sex was homogeneous, with an average age of 11.1 months. The average duration of breastfeeding was 9.4 months, and only 37.1% of the children completed exclusive breastfeeding for six months (Table 1). Colonization by *H. influenzae* was 1.1% (10 isolates) using microbiological methods and 4.1% (37 samples) using PCR. All *H. influenzae* detected by microbiology and/or PCR were NTHi. Of the *H. influenzae* strains identified, 8/10 were found without the presence of other bacteria. Only one child was co-colonized with *H. influenzae* + *S. pneumoniae* and another child with *H. influenzae* + *S. aureus*. Using PCR for *H. influenzae* detection and microbiology for *S. pneumoniae* and *S. aureus*, 22 children (59.5%) were colonized by *H. influenzae* only, 9 (24.3%) by *H. influenzae* + *S. pneumoniae*, and 6 (16.2%) by *H. influenzae* + *S. aureus*. No co-colonization by the three bacteria was found (Table 2).

**Table 1:** Characteristics of the participating children in the study conducted at five hospitals in Lima between 2016 and 2019 (n=888) ^a^

Characteristics		n (%)	Colonization status		p-value
			Not colonized (n=851)	*H. influenzae* (n=37)	
			n (%)	n (%)	
Sex (n=888)					0.978 ^d^
	Female	454 (51.1)	435 (51.1)	19 (51.3)	
	Male	434 (48.9)	416 (48.9)	18 (48.7)	
					
Age (months) ^b^		11.1 ± 6.4	11.1 ± 6.4	11.2 ± 6.8	0.927 ^e^
Age group (months) (n=888)					0.995 ^d^
	≥2 - <6	230 (25.9)	221 (26.0)	9 (24.3)	
	≥6 - <12	226 (25.5)	216 (25.4)	10 (27.0)	
	≥12 - <18	239 (26.9)	229 (26.9)	10 (27.0)	
	≥18 - ≤24	193 (21.7)	185 (21.7)	8 (21.7)	
					
Breastfeeding (months)^b^		9.4 ± 6.1	9.4 ± 6.1	10.1 ± 7.0	0.453 ^e^
Exclusive full breastfeeding (n=878)					0.661 ^d^
	No	552 (62.9)	530 (63.0)	22 (59.5)	
	Yes	326 (37.1)	311 (37.0)	15 (40.5)	
				
Daycare attendance (n=881)					0.483 ^c^
	No	828 (94.0)	794 (94.1)	34 (91.9)	
	Yes	53 (6.0)	50 (5.9)	3 (8.1)	
Lives with other children <6 years (n=873)					0.636 ^d^
	No	528 (60.5)	507 (60.6)	21 (56.8)	
	Yes	345 (39.5)	329 (39.4)	16 (43.2)	
					
Sampling season (n=888)					0.024 ^d^
	Autumn	240 (27.0)	231 (27.1)	9 (24.3)	
	Winter	166 (18.7)	155 (18.2)	11 (29.8)	
	Spring	141 (15.9)	131 (15.4)	10 (27.0)	
	Summer	341 (38.4)	334 (39.3)	7 (18.9)	
Premature (n=864)					0.024 ^d^
	No	682 (78.9)	659 (79.6)	23 (63.9)	
	Yes	182 (21.1)	169 (20.4)	13 (36.1)	
Previous antibiotic use (n=884)					0.899 ^d^
	No	637 (72.1)	610 (72.0)	27 (73.0)	
	Yes	247 (27.9)	237 (28.0)	10 (27.0)	
Previous hospitalization (n=886)					0.506 ^c^
	No	829 (93.6)	793 (93.4)	36 (97.3)	
	Yes	57 (6.4)	56 (6.6)	1 (2.7)	
Mild respiratory disease (n=882)					0.368 ^d^
	No	562 (63.7)	541 (64.0)	21 (56.8)	
	Yes	320 (36.3)	304 (36.0)	16 (43.2)	
Immunized with PCV13 (n=833)					0.577 ^c^
	No	89 (10.7)	84 (10.5)	5 (13.9)	
	Yes	744 (89.3)	713 (89.5)	31 (86.1)	

a Some variables may total fewer than 888 due to missing data.

b Mean (standard deviation).

c Fisher’s exact test.

d Chi-square test (χ²).

e Student’s t-test for equal variances.

PCV13: 13-valent pneumococcal conjugate vaccine

**Table 2:** Identification of co-colonization of *Haemophilus influenzae, Streptococcus pneumoniae,* and S*taphylococcus aureus* using microbiological and molecular methods (PCR)

Colonization Status	***H. influenzae* (microbiology)**	*H. influenzae* (PCR)
	n=10	n=37
*H. influenzae* only	8 (80.0)	22 (59.5)
*H. influenzae* + *S. pneumoniae*	1 (10.0)	9 (24.3)
*H. influenzae* + *S. aureus*	1 (10.0)	6 (16.2)

*H. influenzae* was identified by microbiological and molecular methods. *S. pneumoniae* and *S. aureus* were identified only by microbiological methods.

Of the 10 *H. influenzae* isolates, 8 were biotypes I and 2 were biotype IV (supplementary table 2). Resistance to SXT was detected in 9/10 isolates, while resistance to ampicillin and cefuroxime was found in 3/10 isolates. Furthermore, 2/10 strains showed resistance to azithromycin. No isolates resistant to tetracycline, chloramphenicol, ampicillin-sulbactam, amoxicillin-clavulanic acid, ciprofloxacin, and ceftriaxone were found (Table 3).

**Table 3:** Antimicrobial susceptibility of *Haemophilus influenzae* strains isolated from nasopharyngeal carrier children (N=10) by the disk diffusion method.

Antibiotic	Resistant	Intermediate	Susceptible
	n/N	n/N	n/N
SXT	9/10	1/10	0/10
Ampicillin	3/10	1/10	6/10
Cefuroxime	3/10	3/10	4/10
Azithromycin	2/10	0/10	8/10
Tetracycline	0/10	6/10	4/10
Chloramphenicol	0/10	0/10	10/10
Ampicillin-sulbactam	0/10	0/10	10/10
Amoxicillin-clavulanic acid	0/10	0/10	10/10
Ciprofloxacin	0/10	0/10	10/10
Ceftriaxone	0/10	0/10	10/10

SXT: sulfamethoxazole/trimethoprim

In the bivariate analysis using regressions, premature birth was found to be associated with *H. influenzae* colonization. In the multivariate analysis, the prevalence of being colonized was 2.04 (95% CI: 1.02−4.08; p=0.044) times higher in premature children compared to those born at term (Table 4).

**Table 4:** Factors associated with *Haemophilus influenzae* colonization in nasopharyngeal carrier children ≤ 24 months of age in Lima, Peru.

Characteristics		Bivariate Analysis			Multivariate Analysis^a^		
		PR	95% CI	p value	aPR	95% CI	P value
Sex							
	Female	Ref.			Ref.		
	Male	0.99	0.53−1.86	0.978	1.20	0.61−2.34	0.592
Age group (months)							
	≥2 - <6	Ref.			Ref.		
	≥6 - <12	1.13	0.47−2.73	0.785	1.42	0.47−4.27	0.532
	≥12 - <18	1.07	0.44−2.58	0.882	1.21	0.36−4.04	0.753
	≥18 - ≤24	1.06	0.42−2.69	0.904	1.21	0,39−3.75	0.745
Full exclusive breastfeeding							
	No	Ref.			Ref.		
	Yes	1.15	0.61−2.19	0.661	1.32	0.63−2.75	0.465
Daycare attendance							
	No	Ref.			Ref.		
	Yes	1.38	0.44−4.34	0.584	1.42	0.46−4.39	0.546
Lives with other children <6 years							
	No	Ref.			Ref.		
	Yes	1.17	0.62−2.20	0.636	1.17	0.61−2.26	0.639
Sampling season							
	Autumn	Ref.			Ref.		
	Winter	1.77	0.75−4.17	0.194	1.74	0.64−4.73	0.277
	Spring	1.89	0.79−4.54	0.154	1.79	0.71−4.53	0.219
	Summer	0.55	0.21−1.45	0.225	0.61	0.21−1.77	0.361
Preterm							
	No	Ref.			Ref.		
	Yes	2.12	1.09−4.10	0.026	2.04	1.02−4.08	0.044
Previous antibiotic use							
	No	Ref.			Ref.		
	Yes	0.96	0.47−1.94	0.899	0.84	0.38−1.82	0.652
Previous hospitalization							
	No	Ref.			Ref.		
	Yes	0.40	0.06−2.90	0.367	0.34	0.05−2.06	0.238
Mild respiratory pathology							
	No	Ref.			Ref.		
	Yes	1.34	0.71−2.53	0.370	1.52	0.77−3.00	0.224
Immunized with PCV13							
	No	Ref.			Ref.		
	Yes	0.74	0.30−1.86	0.524	0.41	0.112−1.38	0.148

Bivariate and multivariate analysis using Poisson regression (modified) with robust variances.

PR: Prevalence Ratio. aPR: Adjusted Prevalence Ratio. 95% CI: 95% Confidence Interval. Ref: Reference category. PCV13: Pneumococcal conjugate vaccine 13.

a Adjusted for sex, age group, full exclusive breastfeeding, daycare attendance, living with other children <6 years, sampling season, preterm, previous antibiotic use, previous hospitalization, mild respiratory pathology, and immunized with PCV13.

## DISCUSSION

In this study, we found 4.1% (37/888) nasopharyngeal colonization of *Haemophilus influenzae* in children under 24 months using PCR and 1.1% (10/888) using microbiological methods. All colonized children carried an NTHi strain. 40.5% (15/37) of the *H. influenzae* shared the niche with Streptococcus pneumoniae or Staphylococcus aureus, while 8/10 of the isolates were biotypes I and 2/10 were biotype IV. No resistance to ceftriaxone was found, and only 3/10 isolates showed resistance to ampicillin.

*H. influenzae* continues to reside occasionally in the nasopharynx. Studies have employed PCR as a rapid method with better performance than traditional ones. In 2013, in Peru, a study in a high-Andean locality detected 37.3% nasopharyngeal carriage using PCR and 23.6% using culture in children ≤35 months ^12^. In India, carriage was 41.7% in the pediatric population ^9^, while in Portugal, between 2015-2019, 84.1% carriage was reported in children <6 years attending daycare centers, using real-time PCR ^16^. The differences observed with our study could be explained by the storage of samples at -80 °C for more than 3 years, which could have affected their viability. Furthermore, the low daycare attendance (6%) in our population could contribute to lower exposure, which would lead to a lower carriage frequency, in contrast to the European context where daycare attendance is higher. Factors such as overcrowding and lower Hib vaccination rates are usually associated with greater transmission; however, in our study, these factors do not seem to explain the lower frequency observed, thus methodological and laboratory differences could also be relevant.

Our findings also suggest a coexistence between *H. influenzae* and other bacteria such as S. *pneumoniae* and *S. aureus* with 24.3% (9/37) and 16.2% (6/37), respectively. Some studies report that there is a positive co-existence among nasopharyngeal carriage pathogens ^17^. When these events occur, one of the microorganisms creates the necessary conditions for the colonization of the other.

Regarding serotypes, a study in Belgium collected nasopharyngeal samples from healthy children and children with acute otitis media between 6-30 months of age. They found that 96.4% of the isolates from healthy children were NTHi, while in those with acute otitis media, only 68.1% of the isolates were NTHi ^18^. The change in serotype distribution has also been described in our region. In Paraguay, H. influenzae strains were isolated from children <5 years old with invasive disease between 1999 and 2017, in which it was found that after the introduction of the vaccine in 2004, 36% were Hib isolates, representing a decrease of 28% compared to the pre-vaccine period, while NTHi strains remained stable, being 49% in the pre-vaccine period and 51% in the post-vaccine period, considered to have emerging potential ^10^. These reports agree with our findings, with all isolates being NTHi. This may be because invasive serotypes such as Hib are being displaced by NTHi strains due to the introduction of the Hib conjugate vaccine in various countries ^1^^,^^4^.

On the other hand, the findings on biotypes could be linked to the pathogenicity of certain diseases. In a study in Portugal, biotypes II and III were detected more frequently (42.6% and 26.6%) in children with otitis media, in contrast to healthy children (36.6% and 22.6%) (16). This differs from our results, where 8/10 isolates were biotype I and 2/10 were biotype IV. Also, in Paraguay, based on isolates from patients with invasive and non-invasive disease, 29% was reported for biotype I and 13% for biotype IV ^10^. These findings suggest that biotypes are linked to the clinical presentation of the patient, which could explain the discrepancy with our study, which only included healthy children. However, the high presence of biotype I, previously associated with invasive disease, mainly meningitis ^10^, is particularly relevant. This last point highlights the importance of colonization studies, since it has been proposed that this status could represent an initial stage in the development of invasive disease ^11^.

The antimicrobial susceptibility analysis is limited by the small number of isolates, although the high frequency of resistant strains stands out. A study in Peru (2003-2006) reported resistances of 63% to co-trimoxazole and 38% to azithromycin in children <14 years old with invasive disease ^19^. In China (2016-2019), in children <3 years old with *H. influenzae* infection, 84.3% resistance to SXT and 78% to ampicillin was found in nasopharyngeal isolates. Combining ampicillin with a beta-lactamase inhibitor and cefotaxime was recommended due to resistance mechanisms such as multi-antibiotic efflux pumps (*ydeA* and *norM* genes) ^20^. These measures would optimize treatment and contribute to the initial empirical management of *H. influenzae* infections.

One of the limitations of this study was the cryopreservation time of the samples. Although the STGG transport medium is recommended for the cryopreservation of respiratory pathogens at a temperature of -80 °C, it is preferable to work directly with the sample shortly after being collected. Furthermore, the subculture used in previous studies may have affected the bacterial density of *H. influenzae* and therefore resulted in lower isolation. Likewise, the age of the data (2018-2019) might not represent the current context of H. influenzae colonization in Peru. A more recent evaluation would be necessary to determine possible changes in the distribution of colonization in the pediatric population. Despite these limitations, the present study is relevant because a PCR for *H. influenzae* detection and another for the detection of the serotypes of this pathogen have been adapted and standardized locally. These methods can be used for future surveillance studies, as well as to aid in the diagnosis of patients suspected of having an H. influenzae infection.

In conclusion, *H. influenzae* colonization was infrequent using both microbiological and molecular methods, corresponding entirely to NTHi, with biotype I predominating. Low levels of resistance to ampicillin were found, and no resistance to ceftriaxone was evidenced. Children with a history of premature birth were associated with greater colonization. Understanding the carriage status of *H. influenzae* and associated characteristics is important when invasive infections are suspected. Monitoring serotypes, biotypes, and resistance profiles would contribute to timely diagnosis, reducing empirical treatment and favoring the appropriate selection of antibiotic therapy.
