# Supplementary material for: Distribution of serotypes, biotype, and antibiotic sensitivity of Haemophilus influenzae in nasopharyngeal carrier children in Lima, Peru
Source: Rev Peru Med Exp Salud Publica. 2025 Nov 20;42(4):416–23. doi: 10.17843/rpmesp.2025.424.14767 (PMC12879982; doi:10.17843/rpmesp.2025.424.14767)
Supplement: Supplementary material. — Available in the electronic version of the RPMESP. [file rpmesp-42-04-14767-s001.docx]

**Material Suplementario**

**Tabla suplementaria 1.** Cebadores para la detección molecular de *Haemophilus influenzae* y sus serotipos

| **Gen / Tipo capsular** | **Nombre del *primer*** | **Secuencia 5´a 3´** | **Referencia** |
| --- | --- | --- | --- |
| *Hpd* | F729 | AGA TTG GAA AGA AAC ACA AGA AAA AGA | Peletiri *et al.* |
|  | R819 | CAC CAT CGGCAT ATT TAA CCA CT |  |
| A | a1 | CTA CTC ATT GCA GCA TTT GC | Falla *et al.* |
|  | a2 | GAA TAT GAC CTG ATC TTC TG |  |
| B | b1 | GCG AAA GTG AAC TCT TAT CTC TC |  |
|  | b2 | GCT TAC GCT TCT ATC TCG GTG AA |  |
| C | c1 | TCT GTG TAG ATG ATG GTT CA |  |
|  | c2 | CAG AGG CAA GCT ATT AGT GA |  |
| D | d1 | TGA TGA CCG ATA CAA CCT GT |  |
|  | d1 | TCC ACT CTT CAA ACC ATT CT |  |
| E | e1 | GGT AAC GAA TGT AGT GGT TAG |  |
|  | e2 | GCT TTA CTG TAT AAG TCT AG |  |
| F | f1 | GCT ACT ATC AAG TCC AAA TC |  |
|  | f2 | CGC AAAT TAT GGA AGA AAG CT |  |

Organización Panamericana de la Salud, Instituto Nacional de Salud de Colombia. Programa de vigilancia de los serotipos y resistencia antimicrobiana de Streptococcus pneumoniae y Haemophilus influenzae. Manual de procedimientos. SIREVA II [Internet]. 2004 [citado 20 de enero de 2025]. Disponible en: <https://www3.paho.org/spanish/ad/ths/ev/labs-manual-vigilancia-serotipos.pdf>

Peletiri IC, Ikeh EI, Ayanbimpe GM, Nna E. Molecular detection and characterization of bacteria from CSF samples of patients with suspected cerebrospinal meningitis in parts of northern Nigeria using metagenomic DNA extracts. African Journal of Clinical and Experimental Microbiology. 2 de julio de 2021;22(3):365-76.

Falla TJ, Crook DW, Brophy LN, Maskell D, Kroll JS, Moxon ER. PCR for capsular typing of Haemophilus influenzae. J Clin Microbiol. octubre de 1994;32(10):2382-6.

**Tabla suplementaria 2.** Pruebas bioquímicas empleadas para la determinación de los biotipos de *Haemophilus influenzae* (N=10)

| **Biotipo** | **Pruebas bioquímicas** | | |  | **Total** |
| --- | --- | --- | --- | --- | --- |
|  | **Producción de Indol** | **Descarboxilación de Ornitina** | **Pruebas de Ureasa** |  | **N=10** |
|  |  |  |  |  | **n (%)** |
| I | + | + | + |  | 8 (80,0) |
| II | + | - | + |  | 0 |
| III | - | - | + |  | 0 |
| IV | - | + | + |  | 2 (20,0) |
| V | + | + | - |  | 0 |
| VI | - | + | - |  | 0 |
| VII | + | - | - |  | 0 |
| VIII | - | - | - |  | 0 |

+: Positivo para la prueba indicada

-: Negativo para la prueba indicada
