# Supplementary material for: Distribution of serotypes, biotype, and antibiotic sensitivity of Haemophilus influenzae in nasopharyngeal carrier children in Lima, Peru
Source: Rev Peru Med Exp Salud Publica. 2025 Nov 20;42(4):416–23. doi: 10.17843/rpmesp.2025.424.14767 (PMC12879982; doi:10.17843/rpmesp.2025.424.14767)
Supplement: Translation

# Translation: Distribución de serotipos, biotipo y sensibilidad antibiótica de *Haemophilus influenzae* en niños portadores nasofaríngeos en Lima, Perú

**Authors:** Brayan E. Gonzales, Maria Pinedo-Bardales, Marcela Lopez-Briceño, Edison A. Rivera-Fernandez, David Durand, Erik H. Mercado, Faviola Valdivia, Theresa J. Ochoa

**Affiliations:** 1 Instituto de Medicina Tropical Alexander von Humboldt, Universidad Peruana Cayetano Heredia, Lima, Perú.; 2 Laboratorio de Infecciones Respiratorias Agudas, Centro Nacional de Salud Pública, Instituto Nacional de Salud, Lima, Perú.; 3 Facultad de Medicina, Universidad Peruana Cayetano Heredia, Lima, Perú.

**Keywords:** Vacuna Hib, Haemophilus influenzae, Colonización, Serotipos, Biotipos

## Abstract

Luego de la introducción de la vacuna Hib (*Haemophilus influenzae* tipo b) disminuyó marcadamente la infección invasiva por esta bacteria, sin embargo, aumentó la preocupación por las otras cepas tipificables y no tipificables (NTHi). La colonización previa con este patógeno puede convertirse en una fuente de infección invasiva. Este estudio tuvo como objetivo determinar la colonización por *H. influenzae* en 888 niños sanos menores de 24 meses de edad. La colonización fue 4,1% (37 muestras) mediante métodos moleculares (todas NTHi) y 1,1% (10 aislamientos) por cultivo (8/10 biotipo I y 2/10 biotipo IV). El antecedente de prematuridad se asoció con una mayor colonización en el análisis multivariado. De los aislamientos, 9/10 fueron resistentes a sulfametoxazol/trimetoprima, 3/10 a ampicilina y cefuroxima, 2/10 a azitromicina, y no se observó resistencia a ceftriaxona. Conocer el estado de portación de *H. influenzae* y los patrones locales de resistencia es de utilidad para la terapia antibiótica empírica en infecciones invasivas en las que se sospecha esta bacteria.

## INTRODUCCIÓN

*Haemophilus influenzae* es un cocobacilo gramnegativo que puede causar infecciones como otitis media, sinusitis y neumonía, principalmente en niños, ancianos y pacientes inmunodeprimidos ^1^. Su transmisión ocurre principalmente por contacto con gotículas respiratorias de los portadores. Los neonatos pueden infectarse al entrar en contacto con secreciones genitales infectadas ^2^.

La colonización de *H. influenzae, y otros patógenos respiratorios como Streptococcus pneumoniae*, es común en niños pequeños. Sin embargo, la colonización puede convertirse en una fuente de infección invasiva. Antes de las vacunas contra *S. pneumoniae* y*H. influenzae*tipo b (Hib), las infecciones respiratorias eran la principal causa de mortalidad en niños <5 años ^3^. De los seis serotipos de *H. influenzae* (a, b, c, d, e y f), el serotipo b (Hib) era la principal causa de meningitis en población pediátrica, antes de la introducción de la vacuna Hib en la década de 1990 ^4^^).^ Asimismo, existen cepas sin cápsula, denominadas no-tipificable (NTHi). Aunque el papel de la vacuna Hib en el reemplazo no es claro, las cepas NTHi se han convertido en una causa creciente de enfermedad invasiva ^1^.

Tres décadas después de la introducción de la vacuna Hib en todo el mundo (introducida parcialmente en 1998 y universalmente en 2004 en Perú ^5^), la epidemiología de la enfermedad invasiva por *H. influenzae* ha cambiado sustancialmente ^1^, con un respectivo incremento en otros serotipos ^4^. Del mismo modo, se utilizan ocho biotipos como marcadores epidemiológicos para estudiar la colonización y las cepas patógenas. El biotipo I se asocia con meningitis en niños y mayor resistencia antimicrobiana; los biotipos II y III con infecciones de las vías respiratorias superiores ^6^^-^^8^. Actualmente, la creciente resistencia de *H. influenzae* a betalactámicos, especialmente a penicilina, es motivo de preocupación ^9^^,^^10^.

Las estrategias de prevención y manejo de *H. influenzae* deben basarse en estudios de vigilancia. Sin embargo, estos estudios son escasos en Perú. El objetivo de este estudio fue identificar los principales serotipos y biotipos de *H. influenzae* en niños <24 meses que acuden a establecimientos de salud, así como sus perfiles de resistencia antimicrobiana y factores asociados con el estado de colonización.

**MENSAJE CLAVE**

Motivación para realizar el estudio. Tras la introducción de la vacuna contra Hib, disminuyeron las infecciones invasivas, pero aumentó la preocupación por cepas no tipificables. Este estudio buscó evaluar la colonización por *H. influenzae* en niños peruanos sanos.

Principales hallazgos. La colonización fue 4,1% por métodos moleculares y 1,1% por cultivo, siendo todas no tipificables. Predominó el biotipo I. Solo 3/10 cepas fueron resistentes a ampicilina y no hubo resistencia a ceftriaxona. El nacimiento prematuro del niño se asoció a mayor colonización.

Implicancias para la salud pública. Los hallazgos resaltan la necesidad de vigilancia continua y conocimiento de resistencia local para orientar la terapia empírica y las estrategias de prevención.

## EL ESTUDIO

### Diseño de estudio

Este estudio multicéntrico transversal utilizó datos y muestras del estudio previo sobre portadores de *S. pneumoniae*^11^, el cual incluyó 1000 niños sanos de 2-24 meses de edad atendidos en consultas de pediatría, control de niño sano y vacunación de cinco hospitales nacionales (Hospital Nacional Cayetano Heredia, Instituto Nacional de Salud del Niño Sede Breña, Hospital Nacional Edgardo Rebagliati Martins, Hospital Nacional Docente Madre Niño San Bartolomé y Hospital Nacional Daniel Alcides Carrión) en Lima entre 2018-2019. Se contó con los mismos criterios de inclusión y exclusión que el estudio previo, de los cuales, solo en 888 muestras pertenecían a participantes que habían dado su consentimiento para uso en estudios futuros y se contaba con muestra remanente.

### Colección, transporte y procesamiento de muestras

En el estudio previo, se recolectaron bajo técnica estéril muestras nasofaríngeos de los participantes con un hisopo de Rayon (Puritan®*),* siguiendo las recomendaciones de la Organización Mundial de la Salud ^11^. Las muestras se transportaron en medio STGG (Skim milk, tryptone, glucosa and glycerol) en hielo, manteniendo la cadena de frio y dentro de las 8 horas de su recolección al Laboratorio de Infectología Pediátrica de la Universidad Peruana Cayetano Heredia ^11^. Luego, una alícuota de 200μL se almacenó en STGG a -80 °C para futuros estudios. Este estudio empleó dichas muestras almacenadas y fueron enriquecidas en *Brain Heart Infusion* (BHI) suplementado con 5% de *Haemophilus Test Medium Supplement* (OXOID) durante 4-6 horas a 37 °C en 5% de CO_2_. El cultivo enriquecido se sembró en agar chocolate con bacitracina con las condiciones previamente descritas durante 24 horas ^12^.

### Identificación microbiológica y molecular de *H. influenzae*

Los aislamientos de *H. influenzae* se identificaron mediante morfología de colonia, ausencia de hemólisis, coloración Gram, prueba de catalasa, requerimiento de factores X y V ^13^. Adicionalmente, todos los hisopados nasofaríngeos se evaluaron por reacción en cadera de la polimerasa (PCR) para detectar el gen *Hpd* de *H. influenzae* (tabla suplementaria 1).

### Biotipificación microbiológica y serotipificación molecular

Los biotipos de aislamientos positivos a *H. influenzae* fueron evaluados mediante la pruebas de ureasa, producción de indol y descarboxilación de ornitina ^14^. El ADN de los aislamientos se obtuvo mediante el uso de Chelex®100 y se realizaron una serie de PCR secuenciales para la serotipificación. La primera PCR detecta el gen *Hpd* (confirmación de *H. influenzae*) y las cepas confirmadas pasaron a una PCR con seis pares de *primers* para identificar los tipos capsulares (tabla suplementaria 1) contando con cepas de referencia como control positivo.

### Susceptibilidad antibiótica

La susceptibilidad antibiótica a ampicilina, amoxicilina/ácido clavulánico, ampicilina/sulbactam, cefuroxima, ceftriaxona, cefotaxima, meropenem, levofloxacino, sulfametoxazol/trimetoprima (SXT), azitromicina y cloranfenicol se determinó mediante el método de disco difusión. Para las definiciones de sensibilidad (sensible, intermedio, resistente) se empleó los estándares del Clinical and Laboratory Standard Institute (CLSI) publicados en el documento M100, 33 Ed ^15^.

### Operacionalización de variables

El estudio previó realizó una encuesta al cuidador del niño para recopilar la información demográfica y clínica. Se construyó un diagrama acíclico dirigido (DAG) para seleccionar las variables potencialmente asociadas con la colonización de *H. influenzae*. Estas variables fueron sexo, edad en meses, grupo etario (≥2-<6, ≥6-<12, ≥12-<18 y ≥18-≤24 meses), duración de la lactancia materna en meses, cumplió con la lactancia materna exclusiva de 6 meses, asistencia a guardería, vive con otros niños <6 años, estación de muestreo (otoño, invierno, primavera y verano), nacimiento prematuro, haber usado antibióticos previo a los 3 meses de la toma de muestra, haber estado hospitalización hasta 3 meses previo a la toma de muestra, presencia de al menos una patología respiratoria leve (tos leve, estornudo, rinorrea fiebre entre 38-38,5°C) e inmunizado con PCV13 (Pneumococcal conjugate vaccine 13).

### Análisis estadístico

La descripción de las características y comparación con las frecuencias de portación fueron realizadas mediante la prueba de T de Student (para variables numéricas con distribución normal), Chi cuadrado (χ2) y exacta de Fisher (para variables categóricas). Como medida de asociación se estimó la razón de prevalencias (RP) mediante regresión de Poisson con varianzas robustas. Empleando el criterio epidemiológico, todas las variables potencialmente asociadas con la colonización de *H. influenzae* fueron seleccionadas en el modelo ajustado multivariado. Se consideró intervalos de confianza al 95% (IC 95%) y un valor de p<0,05 como estadísticamente significativo. Todos los análisis se realizaron empleando el paquete estadístico Stata/SE v.18,0.

### Aspectos éticos

El estudio fue registrado en el Sistema Descentralizado de Información y Seguimiento a la Investigación (SIDISI) y evaluado por el Comité Institucional de Ética de la Universidad Peruana Cayetano Heredia. Solo se incluyeron muestras de participantes que habían dado su consentimiento para uso futuro de su muestra en el estudio inicial de portación de neumococo.

## HALLAZGOS

De la muestra incluida, la distribución por sexo fue homogénea, con una edad promedio de 11,1 meses. La lactancia materna promedio fue de 9,4 meses y solo el 37,1% de los niños completó la lactancia exclusiva de seis meses (tabla 1). La colonización por *H. influenzae* fue del 1,1% (10 aislamientos) usando métodos microbiológicos y del 4,1% (37 muestras) usando PCR. Todos los *H. influenzae* detectados por microbiología y/o PCR fueron NTHi. De las cepas de *H. influenzae* identificadas, 8/10 se encontraron sin presencia de otras bacterias. Solo un niño estaba co-colonizado con *H. influenzae* + *S. pneumoniae* y otro niño con *H. influenzae + S. aureus*. Usando PCR para la detección de *H. influenzae* y microbiología para *S. pneumoniae* y *S. aureus*, 22 niños (59,5%) fueron colonizados solo por *H. influenzae*, 9 (24,3%) por *H. influenzae* + *S. pneumoniae*, y 6 (16,2%) por *H. influenzae + S. aureus*. No se encontró co-colonización por las tres bacterias (tabla 2).

**Tabla 1:** Características de los niños participantes del estudio realizado en cinco hospitales de Lima entre los años 2016 y 2019 (n=888) ^a^

Características		n (%)	Estado de colonización		Valor de p
			No colonizado (n=851)	*H. influenzae* (n=37)	
			n (%)	n (%)	
Sexo (n=888)					0,978 ^d^
	Femenino	454 (51,1)	435 (51,1)	19 (51,3)	
	Masculino	434 (48,9)	416 (48,9)	18 (48,7)	
					
Edad (meses) ^b^		11,1 ± 6,4	11,1 ± 6,4	11,2 ± 6,8	0,927 ^e^
Grupo etario (meses) (n=888)					0,995 ^d^
	≥2 - <6	230 (25,9)	221 (26,0)	9 (24,3)	
	≥6 - <12	226 (25,5)	216 (25,4)	10 (27,0)	
	≥12 - <18	239 (26,9)	229 (26,9)	10 (27,0)	
	≥18 - ≤24	193 (21,7)	185 (21,7)	8 (21,7)	
					
Lactancia materna (meses) ^b^		9,4 ± 6,1	9,4 ± 6,1	10,1 ± 7,0	0,453 ^e^
Lactancia materna exclusiva completa (n=878)					0,661 ^d^
	No	552 (62,9)	530 (63,0)	22 (59,5)	
	Si	326 (37,1)	311 (37,0)	15 (40,5)	
				
Asistencia a guardería (n=881)					0,483 ^c^
	No	828 (94,0)	794 (94,1)	34 (91,9)	
	Sí	53 (6,0)	50 (5,9)	3 (8,1)	
Vive con otros niños <6 años (n=873)					0,636 ^d^
	No	528 (60,5)	507 (60,6)	21 (56,8)	
	Sí	345 (39,5)	329 (39,4)	16 (43,2)	
					
Estación de muestreo (n=888)					0,024 ^d^
	Otoño	240 (27,0)	231 (27,1)	9 (24,3)	
	Invierno	166 (18,7)	155 (18,2)	11 (29,8)	
	Primavera	141 (15,9)	131 (15,4)	10 (27,0)	
	Verano	341 (38,4)	334 (39,3)	7 (18,9)	
Prematuro (n=864)					0,024 ^d^
	No	682 (78,9)	659 (79,6)	23 (63,9)	
	Sí	182 (21,1)	169 (20,4)	13 (36,1)	
Uso de antibióticos previo (n=884)					0,899 ^d^
	No	637 (72,1)	610 (72,0)	27 (73,0)	
	Si	247 (27,9)	237 (28,0)	10 (27,0)	
Hospitalización previa (n=886)					0,506 ^c^
	No	829 (93,6)	793 (93,4)	36 (97,3)	
	Sí	57 (6,4)	56 (6,6)	1 (2,7)	
Patología respiratoria leve (n=882)					0,368 ^d^
	No	562 (63,7)	541 (64,0)	21 (56,8)	
	Sí	320 (36,3)	304 (36,0)	16 (43,2)	
Inmunizado con PCV13 (n=833)					0,577 ^c^
	No	89 (10,7)	84 (10,5)	5 (13,9)	
	Sí	744 (89,3)	713 (89,5)	31 (86,1)	

a Algunas variables pueden sumar menos de 888 por datos faltantes.

b Media (desviación estándar).

c Prueba exacta de Fisher.

d Prueba Chi cuadrado (χ2)

e T de Student de varianzas iguales

PCV13: Pneumococcal conjugate vaccine 13

**Tabla 2:** Identificación de co-colonización de *Haemophilus influenzae*, *Streptococcus pneumoniae* y *Staphylococcus aureus* usando métodos microbiológicos y moleculares (PCR).

Estado de colonización	*H. influenzae* (microbiología)	*H. influenzae* (PCR)
	n=10	n=37
Solo *H. influenzae*	8 (80,0)	22 (59,5)
*H. influenzae* + *S. pneumoniae*	1 (10,0)	9 (24,3)
*H. influenzae* + *S. aureus*	1 (10,0)	6 (16,2)

*H. influenzae* fue identificado por métodos microbiológicos y moleculares. *S. pneumoniae* y *S. aureus* fueron identificados solo por métodos microbiológicos.

De los 10 *H. influenzae* aislados, 8 fueron biotipo I y 2 del biotipo IV (tabla suplementaria 2). La resistencia a SXT se detectó en 9/10 aislamientos, mientras que la resistencia a ampicilina y cefuroxima se encontró en 3/10 aislamientos. Además, 2/10 cepas mostraron resistencia a azitromicina. No hubo aislamientos resistentes a tetraciclina, cloranfenicol, ampicilina-sulbactam, amoxicilina-ácido clavulánico, ciprofloxacino y ceftriaxona (tabla 3).

**Tabla 3:** Susceptibilidad antimicrobiana por el método de disco difusión de cepas de *Haemophilus influenzae* aislados de niños portadores nasofaríngeos (N=10).

Antibiótico	Resistente	Intermedio	Sensible
	n/N	n/N	n/N
SXT	9/10	1/10	0/10
Ampicilina	3/10	1/10	6/10
Cefuroxima	3/10	3/10	4/10
Azitromicina	2/10	0/10	8/10
Tetraciclina	0/10	6/10	4/10
Cloranfenicol	0/10	0/10	10/10
Ampicilina-sulbactam	0/10	0/10	10/10
Amoxicilina-ácido clavulánico	0/10	0/10	10/10
Ciprofloxacino	0/10	0/10	10/10
Ceftriaxona	0/10	0/10	10/10

SXT: sulfametoxazol/trimetoprima

En el análisis bivariado mediante regresiones, se encontró que el nacimiento prematuro se asoció con la colonización por *H. influenzae*. En el análisis multivariado, la prevalencia de estar colonizado fue 2,04 (IC 95%: 1,02−4,08; p=0,044) veces mayor en los niños prematuros en comparación con aquellos nacidos a termino (tabla 4).

**Tabla 4:** Factores asociados a la colonización por *Haemophilus influenzae* en niños portadores nasofaríngeos ≤ 24 meses de edad en Lima, Perú.

Características		Análisis bivariado			Análisis multivariado ^a^		
		RP	IC 95%	Valor de p	RPa	IC 95%	Valor de p
Sexo							
	Femenino	Ref.			Ref.		
	Masculino	0,99	0,53−1,86	0,978	1,20	0,61−2,34	0,592
Grupo etario (meses)							
	≥2 - <6	Ref.			Ref.		
	≥6 - <12	1,13	0,47−2,73	0,785	1,42	0,47−4,27	0,532
	≥12 - <18	1,07	0,44−2,58	0,882	1,21	0,36−4,04	0,753
	≥18 - ≤24	1,06	0,42−2,69	0,904	1,21	0,39−3,75	0,745
Lactancia materna exclusiva completa							
	No	Ref.			Ref.		
	Sí	1,15	0,61−2,19	0,661	1,32	0,63−2,75	0,465
Asistencia a guardería							
	No	Ref.			Ref.		
	Sí	1,38	0,44−4,34	0,584	1,42	0,46−4,39	0,546
Vive con otros niños <6 años							
	No	Ref.			Ref.		
	Sí	1,17	0,62−2,20	0,636	1,17	0,61−2,26	0,639
Estación de muestreo							
	Otoño	Ref.			Ref.		
	Invierno	1,77	0,75−4,17	0,194	1,74	0,64−4,73	0,277
	Primavera	1,89	0,79−4,54	0,154	1,79	0,71−4,53	0,219
	Verano	0,55	0,21−1,45	0,225	0,61	0,21−1,77	0,361
Prematuro							
	No	Ref.			Ref.		
	Sí	2,12	1,09−4,10	0,026	2,04	1,02−4,08	0,044
Uso de antibióticos previo							
	No	Ref.			Ref.		
	Sí	0,96	0,47−1,94	0,899	0,84	0,38−1,82	0,652
Hospitalización previa							
	No	Ref.			Ref.		
	Sí	0,40	0,06−2,90	0,367	0,34	0,05−2,06	0,238
Patología respiratoria leve							
	No	Ref.			Ref.		
	Sí	1,34	0,71−2,53	0,370	1,52	0,77−3,00	0,224
Inmunizado con PCV13							
	No	Ref.			Ref.		
	Sí	0,74	0,30−1,86	0,524	0,41	0,112−1,38	0,148

Análisis bivariado y multivariado usando regresión de Poisson (modificado) con varianzas robustas.

RP: razón de prevalencias. RPa: razón de prevalencias ajustado. IC 95%: intervalo de confianza 95%. Ref: categoría de referencia. PCV13: Pneumococcal conjugate vaccine 13.

a Ajustado por sexo, grupo etario, lactancia materna exclusiva completa, asistencia a guardería, vive con otros niños <6 años, estación de muestreo, prematuro, uso de antibióticos previo, hospitalización previa, patología respiratoria leve, inmunizado con PCV13.

## DISCUSIÓN

En este estudio encontramos 4,1% (37/888) de colonización nasofaríngea de *Haemophilus influenzae* en niños menores de 24 meses empleando PCR y 1,1% (10/888) empleando métodos microbiológicos. Todos los niños colonizados portaban una cepa NTHi. El 40,5% (15/37) de los *H. influenzae* compartían el nicho con *Streptococcus pneumoniae* o *Staphylococcus aureus*, mientras que 8/10 de los aislamientos fueron del biotipo I y 2/10 fueron del biotipo IV. No se encontró resistencia a ceftriaxona y solo 3/10 aislamientos presentaron resistencia a ampicilina.

*H. influenzae* continúa residiendo ocasional en la nasofaringe. Estudios han empleado PCR como método rápido con mejor rendimiento que los tradicionales. En 2013, en Perú, un estudio en una localidad altoandina detectó 37,3% de portación nasofaríngea empleando PCR y 23,6% empleando cultivo en niños ≤35 meses ^12^. En la India, la portación fue 41,7% en población pediátrica ^9^, mientras que en Portugal, entre 2015-2019, se reportó 84,1% de portación en niños <6 años que asistían a guarderías, empleando PCR en tiempo real ^16^. Las diferencias observadas con nuestro estudio podrían explicarse por la conservación de muestras a -80 °C por más de 3 años, lo que podría haber afectado su viabilidad. Además, la baja asistencia a guarderías (6%) en nuestra población podría contribuir a una menor exposición lo que llevaría a una menor frecuencia de portación, en contraste con el contexto europeo donde la asistencia a guarderías es más alta. Factores como el hacinamiento y las menores tasas de vacunación contra Hib suelen asociarse a una mayor transmisión; sin embargo, en nuestro estudio estos factores no parecen explicar la menor frecuencia observada, por lo tanto, diferencias metodológicas y de laboratorio también podrían ser relevantes.

Nuestros hallazgos también sugieren una coexistencia entre *H. influenzae* y otras bacterias como *S. pneumoniae* y *S. aureus* con el 24,3% (9/37) y 16,2% (6/37), respectivamente. Algunos estudios informan que existe una coexistencia positiva entre patógenos de portación nasofaríngea ^17^. Cuando ocurren estos eventos, uno de los microorganismos propicia las condiciones necesarias para la colonización del otro.

Con respecto a los serotipos, un estudio en Bélgica colectó muestras nasofaríngeas de niños sanos y niños con otitis aguda entre 6-30 meses de edad. Encontraron que el 96,4% los aislamientos de niños sanos fueron NTHi, mientras que en los que tenían otitis aguda solo el 68,1% de los aislamientos fueron NTHi ^18^. El cambio en la distribución de serotipos también ha sido descrito en nuestra región. En Paraguay se aislaron cepas de *H. influenzae* de niños <5 años con enfermedad invasiva entre 1999 a 2017, en los cuales se encontró que luego de la introducción de la vacuna en el año 2004, el 36% fueron aislamientos Hib, representando una disminución de un 28% con respecto al periodo prevacunal, mientras que las cepas NTHi se mantuvieron estables, siendo 49% en el periodo prevacunal y 51% en el periodo posvacunal, considerándose con potencial emergente ^10^. Estos reportes concuerdan con nuestros hallazgos siendo todos los aislamientos NTHi. Esto puede deberse a que los serotipos invasivos como Hib están siendo desplazados por las cepas NTHi debido a la introducción de la vacuna conjugada Hib en diversos países ^1^^,^^4^.

Por otro lado, los hallazgos sobre los biotipos podrían vincularse con la patogenicidad de ciertas enfermedades. En un estudio en Portugal, los biotipos II y III se detectaron con mayor frecuencia (42,6% y 26,6%) en niños con otitis media, en contraste con niños sanos (36,6% y 22,6%) ^16^. Esto difiere de nuestros resultados, donde 8/10 aislamientos fueron del biotipo I y 2/10 del biotipo IV. Asimismo, en Paraguay, basado en aislamientos de pacientes con enfermedad invasiva y no invasiva, se reportó 29% para biotipo I y 13% para biotipo IV ^10^. Estos hallazgos sugieren que los biotipos se vinculan con el cuadro clínico del paciente, lo cual podría explicar la discrepancia con nuestro estudio, en el que solo se incluyeron niños sanos. Sin embargo, resulta particularmente relevante la alta presencia del biotipo I, previamente asociado con enfermedad invasiva, principalmente meningitis ^10^. Esto último resalta la importancia de estudios de colonización, ya que se ha propuesto que dicho estado podría representar una etapa inicial en el desarrollo de enfermedad invasiva ^11^.

El análisis de susceptibilidad antimicrobiana se ve limitado por el reducido número de aislamientos, aunque destaca la alta frecuencia de cepas resistentes. Un estudio en Perú (2003-2006) reportó resistencias del 63% a cotrimoxazol y 38% a azitromicina en niños <14 años con enfermedad invasiva ^19^. En China (2016-2019), en niños <3 años con infección por *H. influenzae*, se encontró resistencia del 84,3% a SXT y 78% a ampicilina en aislamientos nasofaríngeas. Se recomendó combinar ampicilina con inhibidor de betalactamasa y cefotaxima debido a mecanismos de resistencia como bombas de eflujo de múltiples antibióticos (genes *ydeA* y *norM*) ^20^. Estas medidas optimizarían el tratamiento y contribuyen al manejo empírico inicial de infecciones por *H. influenzae*.

Una de las limitaciones de este estudio, fue el tiempo de criopreservación de las muestras. Si bien el medio de transporte STGG es recomendado para la criopreservación de patógenos respiratorios a una temperatura de -80 °C, es preferible trabajar directamente con la muestra a poco tiempo de haber sido colectada. Además, el subcultivo utilizado en estudios anteriores, pueden haber afectado la densidad bacteriana de *H. influenzae* y por ende un menor aislamiento. Asimismo, la antigüedad de los datos (2018-2019) podría no representar el contexto actual de la colonización por *H. influenzae* en Perú. Sería necesario una evaluación más reciente para determinar posibles cambios en la distribución de colonización en la población pediátrica. Pese a estas limitaciones, el presente estudio es relevante porque se ha adaptado y estandarizado a nivel local un PCR para detección de *H. influenzae* y otro para la detección de los serotipos de este patógeno. Estos métodos podrán ser utilizados para futuros estudios de vigilancia, así como para ayudar en el diagnóstico de pacientes con sospecha de infección por *H. influenzae.*

En conclusión, la colonización por *H. influenzae* fue poco frecuente tanto al emplear métodos microbiológicos como moleculares, correspondiendo en su totalidad por NTHi, predominando el biotipo I. Se encontraron bajos niveles de resistencia a ampicilina y no se evidenció resistencia a ceftriaxona. Los niños con antecedente de nacimiento prematuro se asociaron con una mayor colonización. Conocer el estado de portación de *H. influenzae* y las características asociadas es importante ante la sospecha de infecciones invasivas. El monitoreo de serotipos, biotipos y perfiles de resistencia contribuiría a un diagnóstico oportuno, disminuyendo el tratamiento empírico y favoreciendo la selección adecuada de la terapia antibiótica.
